# Kinetics of the Interactions between Copper and Amyloid‐β with FAD Mutations and Phosphorylation at the N terminus

**DOI:** 10.1002/cbic.201600255

**Published:** 2016-08-02

**Authors:** Paul Girvan, Toru Miyake, Xiangyu Teng, Thomas Branch, Liming Ying

**Affiliations:** ^1^ Molecular MedicineNational Heart and Lung InstituteImperial College LondonExhibition RoadLondonSW7 2AZUK; ^2^ Institute of Chemical BiologyImperial College LondonExhibition RoadLondonSW7 2AZUK; ^3^ Department of ChemistryImperial College LondonExhibition RoadLondonSW7 2AZUK; ^4^ Faculty of MedicineTokyo Medical and Dental University1-5-45 YushimaBunkyoTokyo113-0034Japan

**Keywords:** amyloid beta-peptides, copper, fluorescence spectroscopy, kinetics, reaction mechanism

## Abstract

Mutations and post‐translational modifications of amyloid‐β (Aβ) peptide in its N terminus have been shown to increase fibril formation, yet the molecular mechanism is not clear. Here we investigated the kinetics of the interactions of copper with two Aβ peptides containing Familial Alzheimer's disease (FAD) mutations (English (H6R) and Tottori (D7N)), as well as with Aβ peptide phosphorylated at serine 8 (pS8). All three peptides bind to copper with a similar rate as the wild‐type (wt). The dissociation rates follow the order pS8>H6R>wt>D7N; the interconversion between the two coordinating species occurs 50 % faster for H6R and pS8, whereas D7N had only a negligible effect. Interestingly, the rate of ternary complex (copper‐bridged heterodimer) formation for the modified peptides was significantly faster than that for wt, thus leading us to propose that FAD and sporadic AD might share a kinetic origin for the enhanced oligomerisation of Aβ.

## Introduction

Alzheimer's disease (AD) is the most common form of dementia, currently affecting 46 million people worldwide.^1^ The roles of amyloid‐β (Aβ) aggregates in the initiation of AD are extensively documented. Aβ peptides are proteolytically processed by secretases into fragments of amyloid precursor protein (APP),^2^ with peptides of 40 (Aβ40) and 42 (Aβ42) residues as the predominant species.

Familial Alzheimer's disease (FAD) is an early‐onset AD; an individual has a heritable mutation that causes the onset of clinical symptoms earlier in life (under the age of 65). Although FAD accounts for only a small proportion of AD cases, it is regarded as useful to study, in order to help better understand the disease as a whole.^3^

FAD autosomally dominant mutations have been identified in three proteins related to Aβ production: presenilin‐1 and ‐2 and APP. An extensive review of these FAD mutations is provided by Weggen and Beher;^3^ in summary, most of these mutations either cause an increase in the production of Aβ or alter the Aβ40/Aβ42 ratio. This alteration is the rationale behind developing AD earlier in life. Many of the APP mutations occur at or around the cleavage sites for β‐secretase or, more commonly, γ‐secretase; thus, aetiology involving altered cleavage is likely. However some mutations, such as the substitutions English (H6R, age of onset 55 years)^4^ and Tottori (D7N, 60 years),^5^ are distal from the cleavage sites. Consequently, a different mechanism could account for developing AD from these mutations.

In vitro studies of Aβ40 and Aβ42 with either the H6R or the D7N mutation have shown that these two mutations do not affect the production of Aβ, but alter Aβ assembly at its earliest stages, as well as monomer folding and oligomerisation processes.^6^, ^7^ More recent experimental work with ion‐mobility‐based MS indicated that structural changes in the monomers are reflected in the oligomers.^8^ Molecular dynamics simulations have shed light on how the mutations affect the peptide properties. For H6R, the reduction in the net charge was found to be a major contributor to enhancing aggregation; however, the mutation also caused an increase in hydrophobicity, and this compounded the issue.^9^ D7N was shown to change the fold and salt‐bridge network within the peptide.^10^ Furthermore, these mutations can significantly increase the solvation free energy and thus enhance the aggregation of Aβ monomers.^11^

Apart from the two prevalent species (Aβ40 and Aβ42), a variety of post‐translationally modified variants have been identified;^12^ these include truncation, pyroglutamination, metal induced oxidation and phosphorylation,^13^, ^14^ and they enhance Aβ aggregation.^15^ Phosphorylation regulates the structure and function of many proteins in healthy and diseased tissue, and particularly of intrinsically disordered proteins.^16^ In AD, Tau is abnormally hyperphosphorylated, although the role of this is still under debate.^17^ Aβ has potential phosphorylation sites at Ser8, Ser26 and Tyr10, and can undergo phosphorylation by protein kinase A and cdc2 in vitro.^18^ Walter and co‐workers showed that Aβ is phosphorylated at Ser8 by extracellular protein kinase A, thus promoting the formation of toxic oligomer in a mouse model of AD.^19^, ^20^ Ser8 phosphorylation promotes zinc‐induced dimerisation of Aβ and stabilises the amyloid deposits.^21^, ^22^ These studies suggest that phosphorylated Aβ could be one of the important species in the formation and stabilisation of neurotoxic aggregates, and might be targeted for AD therapy and diagnosis.

It is widely accepted that oligomers are predominantly responsible for the neuronal toxicity of Aβ, and that metal ions play important roles in the oligomerisation.^23^, ^24^ The English and Tottori mutations occur in the Aβ N terminus, a region that has been identified as able to bind to neurometals, such as zinc and copper. The H6R mutation facilitates zinc‐induced dimerisation of the N terminus;^25^ however, here we focus exclusively on copper, as it has much stronger interactions with Aβ.^26^ Furthermore, its interactions with wild‐type Aβ (wt‐Aβ) has been linked with an increase in oligomerisation/fibrillisation, as well as being a source of oxidative damage.^27^ The coordination environment of copper bound to Aβ has been solved by EPR for both wt‐Aβ and mutants H6R and D7N.^28^, ^29^ His6 was identified as a key coordinating ligand for copper in wt‐Aβ, and H6R and D7N mutations alter the binding equilibrium (particularly for H6R as it lacks the coordinating His6 residue).^28^, ^29^ Phosphorylation is fundamentally an oxidation process, thus oxidative stress would enhance the phosphorylation of Aβ in a way similar to that of Tau protein. Copper coordination to phosphorylated Aβ has not been investigated. As Ser8 is not directly coordinated with copper, phosphorylation would have only a minor effect on binding affinity.

Chemical kinetics has provided unprecedented mechanistic insights into amyloid fibril assembly and inhibition.^30^, ^31^ In a highly dynamic system such as the brain, kinetics might dominate over thermodynamics in the interactions between Aβ and metal ions.^32^ Here we report the effects of two FAD mutations on the kinetics of Aβ reactions involving copper, along with a post‐translational modification in the same region. We found that these changes modulate the kinetics of interconversion between two coordination modes and enhance copper‐bridged dimer formation; this might subsequently alter early oligomerisation, such as nucleation, which controls the overall fibrillisation rate.

## Results and Discussion

In order to study the effects of H6R and D7N mutations and Ser8 phosphorylation (pS8) on the binding kinetics of copper to Aβ we employed stopped‐flow techniques, by using the quenching properties of copper on a bright fluorophore covalently attached to Aβ.^33^ Improvements in S/N were achieved by replacing the excitation lamp with a fibre‐coupled diode laser. This facilitated a detailed kinetic study of the interactions between copper and Aβ, thus affording mechanistic insight into the roles mutations and post‐translational modifications might have in FAD and AD.

Except where stated otherwise, truncated Aβ16(H6R), Aβ16(D7N) and Aβ16(pS8) were used, with Lys16 labelled with HiLyte Fluor 488. The first 16 residues contain the metal‐binding site and are a good mimic of the full‐length sequence for the study of monomeric Aβ–metal ion interactions.^34^

**Figure 1:**
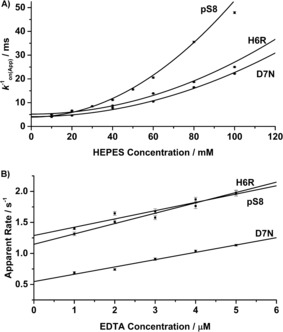
Kinetics of Cu^2+^ binding to Aβ and dissociation from Aβ. A) The binding rate of Cu^2+^ (400 nm) to Aβ (20 nm) depends on HEPES concentration. B) Apparent reaction rate of Aβ**⋅**Cu complex (50 nm) for various concentrations of EDTA.

We first studied the effects of H6R, D7N and pS8 on the Cu^2+^ binding rate constant. In various concentrations of HEPES buffer at 298 K, 20 nm Aβ was treated with 400 nm Cu^2+^ in order to establish the HEPES‐independent Cu^2+^ binding rate constant, *k*_on_ (Figure 1 A; raw data in Figure S2 in the Supporting Information). The data were empirically fitted with a parabola centred at zero, and the intercepts were used to derive the HEPES‐independent rate constants to be 4.8(3)×10^8^ m ^−1^ s^−1^, 6.1(3)×10^8^ m ^−1^ s^−1^ and 6.4(4)×10^8^ m ^−1^ s^−1^ for Aβ16(H6R), Aβ16(D7N) and Aβ16(pS8), respectively. These values are very close to the value previously determined for wt‐Aβ16 (5.0(2)×10^8^ m ^−1^ s^−1^),^33^ thus suggesting that H6R, D7N and pS8 have little impact on the rate of copper binding.

In order to determine the rate at which Cu^2+^ dissociates from Aβ, the complex (50 nm) was treated with low‐micromolar EDTA (Figure 1 B). Extrapolation to zero EDTA yielded apparent dissociation rates of 1.15(5) s^−1^, 0.55(3) s^−1^ and 1.29(5) s^−1^ for Aβ16(H6R), Aβ16(D7N) and Aβ16(pS8) respectively. The second‐order rate constants for the reaction with EDTA were determined from the slopes: 1.7(2)×10^5^ m ^−1^ s^−1^, 1.2(1)×10^5^ m ^−1^ s^−1^ and 1.3(2)×10^5^ m ^−1^ s^−1^ respectively. This is more than twice the value for wt‐Aβ (0.55(3)×10^5^ m ^−1^ s^−1^),^33^ thus suggesting that it is much easier for the Cu^2+^ bound to Aβ(H6R), Aβ(D7N) or Aβ16(pS8) to be attacked by a second ligand compared to the native peptide. This potentially results in more facile copper‐bridged dimer formation.

**Figure 2:**
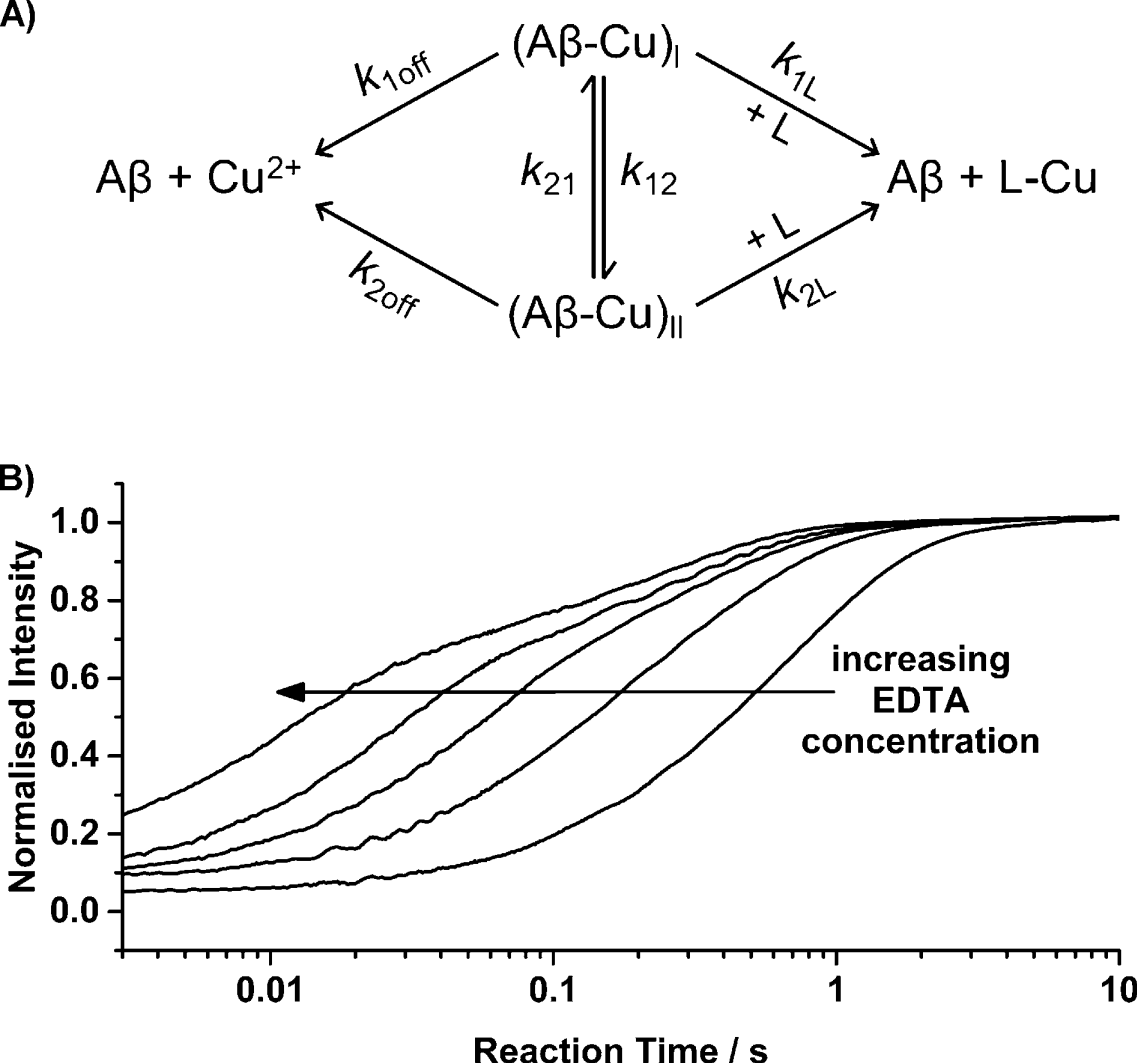
Kinetics of interconversion between the two components of mutant and phosphorylated Aβ**⋅**Cu complexes can be probed by their reactions with EDTA. A) Reaction model to which the data were fitted. B) Raw kinetic data of the reaction between Aβ⋅Cu and 5, 50, 150, 300 and 700 μm EDTA.

**Figure 3:**
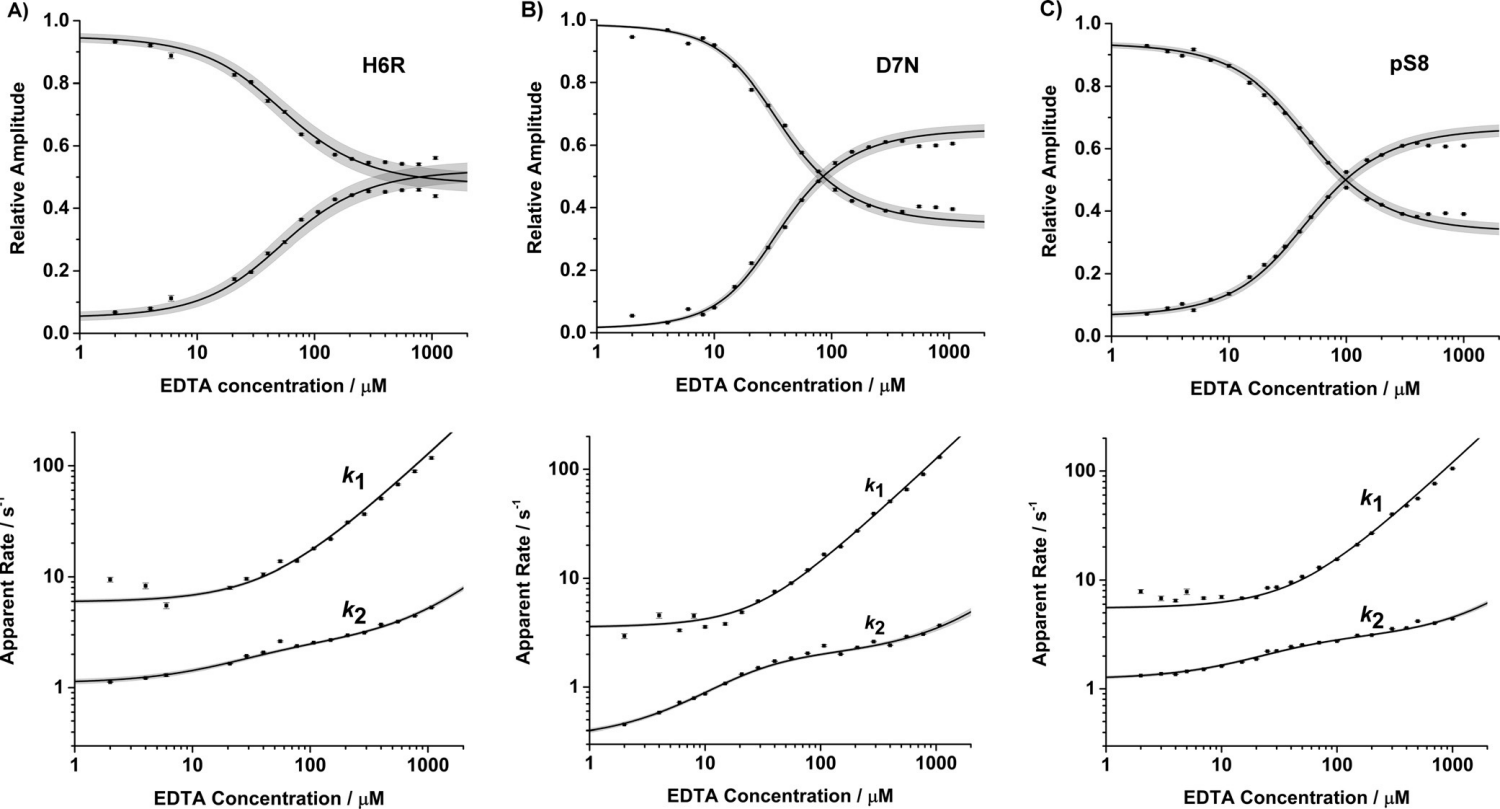
Amplitudes (top) and apparent rates (bottom) for the two phases of the reaction of Aβ**⋅**Cu complexes with EDTA for A) Aβ(H6R), B) Aβ(D7N) and C) Aβ(pS8). The solid lines are the fittings to the reaction scheme. Error boundaries (1*σ*) are shaded. In the top panels, the phases with decreasing amplitude correspond to apparent rate *k*_1_; the phases with increasing amplitude correspond to apparent rate *k*_2_.

Next, the kinetics of interconversion between the two main Aβ**⋅**Cu coordination modes^29^ at physiological pH were investigated. The two main coordination modes of Aβ**⋅**Cu differ in their protonation state and are often referred to as Component **I** (for the protonated species) and Component **II** (for the deprotonated species). Aβ and Cu^2+^ were mixed to generate the 1:1 stoichiometry complex (50 nm), then treated with excess EDTA (2–1000 μm). The data were fitted to the simplest symmetrical model for the interaction of Aβ and copper to determine the kinetic parameters (Figure 2 A; details of the model and data fitting are as previously reported,^33^ summary in the Supporting Information). Representative kinetic raw data are shown in Figures 2 B and S3. Two phases are clearly visible at higher EDTA concentrations. Apparent amplitudes and rates derived from the raw data, as well as fitted curves and error boundaries (1*σ*) are shown in Figure 3. The kinetic parameters derived from the fits are summarised in Table 1, with values for wt‐Aβ shown for comparison.

**Table 1:** Kinetic parameters derived from the reactions of Aβ**⋅**Cu complexes with EDTA.

	Aβ16(H6R)	Aβ16(D7N)	Aβ16(pS8)	wt‐Aβ16^33^
*k*_1off_ [s^−1^]	2.6(1)	0.57(2)	2.25(6)	0.8(1)
*k*_1L_ [10^5^ m ^−1^ s^−1^]	1.23(4)	1.23(3)	1.16(2)	1.3(1)
*k*_12_ [s^−1^]	1.9(2)	1.24(8)	1.48(9)	0.9(2)
*k*_2off_ [s^−1^]	n.d.^[a]^	n.d.^[a]^	n.d.^[a]^	n.d.^[a]^
*k*_2L_ [10^3^ m ^−1^ s^−1^]	2.7(2)	1.4(2)	1.6(1)	1.48(6)
*k*_21_ [s^−1^]	2.54(7)	2.06(6)	3.01(6)	2.22(3)

[a] Too slow to be determined.

Neither mutation nor the phosphorylation had much effect on the rate of Cu^2+^ binding. A slight increase upon phosphorylation can be attributed to the extra negative charge from the phosphate group, thus enhancing the electrostatic attraction between Cu^2+^ and the peptide. Surprisingly, there was virtually no difference in the binding rate with H6R, even though His6 has been identified as a coordinating ligand in wt‐Aβ. Conversely, the rate of copper dissociation (*k*_1off_) from Component **I** for the mutants and phosphorylated peptide differs from wild‐type: D7N dissociated slightly slower, whereas H6R and pS8 dissociated over two times faster. This is reflected in the different dissociation constants and suggests that both the H6R and pS8 peptides bind copper much more weakly than either wt or D7N. This observation allows new mechanistic insight into Cu^2+^ binding and suggests that although His6 is needed to produce a stable complex, its absence does not affect the rate of initial binding. The faster dissociation of Cu from pS8 Aβ is somewhat surprising, and indicates that the extra negative charge from phosphorylation is not a determinant for Cu^2+^ binding. The reaction with the Component **I** coordination mode is of more interest than Component **II** as it is two orders of magnitude faster, and so the reaction of copper‐bridged ternary complex formation likely proceeds through Component **I**. The two FAD mutations and pS8 have almost the same rate constants, compared to the wt, thus suggesting that Component **I** coordination species is insensitive to a strong Cu^2+^ ligand such as EDTA for heterodimer formation. Interconversion between the two coordinating species (*k*_12_ and *k*_21_) occurs 50 % faster for H6R and pS8, but D7N only has minimal effect.

The relative amplitude graphs (Figure 3 top) at high EDTA concentrations, where the bimolecular reactions are much faster than their interconversion between the two coordination modes, gives an indication of the distribution of Aβ**⋅**Cu^2+^ Components **I** and **II** species present under equilibrium. Component **I** is dominant for both D7N and pS8. As the experiments reported here were carried out at pH 7.5 the observation for D7N is in excellent agreement with a previous value for p*K*_a_ (7.7).^29^ The p*K*_a_ values were calculated from the relationship p*K*_a_=pH+log(*k*_21_/*k*_12_); the rate constants *k*_12_ and *k*_21_ derived from the fitting are listed in Table 1. The experimentally determined p*K*_a_ values were 7.62(4), 7.72(3) and 7.81(3) for Aβ(H6R), Aβ(D7N) and Aβ(pS8), respectively. The p*K*_a_ for Aβ(pS8) has not previously been reported. The discrepancy between the value for H6R and a previously reported value by EPR (7.2)^29^ could be due to the less good fit of the Aβ(H6R) data to the model, thus suggesting that the Aβ(H6R) system might be more complex than the simplest two component interconverting model described. The discrepancy could also be due to a difference in temperature (EPR measurements were carried out at 110 K).

Metal ions such as copper and zinc are involved in Aβ aggregation.^35^ In the absence of metal ions, Aβ peptide forms dimers at a rate approximately 10^2^–10^3^ m ^−1^ s^−1^.^36^ It was estimated recently that the dimerisation rate of Aβ increased by more than two orders of magnitude in the presence of Cu^2+^.^33^ Furthermore, a single‐molecule force spectroscopy study suggested that Cu^2+^ acts as a bridge between the two peptide molecules, thereby increasing the stability of the peptide–peptide complex.^37^ To assess the effect of FAD mutations and Ser8 phosphorylation on the kinetics of copper‐bridged dimerisation of Aβ, the formation rate of the Aβ⋅Cu^2+^⋅Aβ ternary complex was determined.

**Figure 4:**
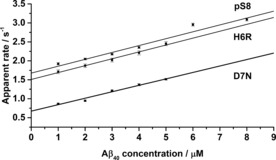
Kinetics of mutated Aβ16**⋅**Cu complexes reacting with Aβ40 bearing the same mutations as well as phosphorylated Aβ16**⋅**Cu reacting with wt‐Aβ40.

Aβ**⋅**Cu^2+^ complexes of H6R, D7N or pS8, were pre‐formed and then treated with unlabelled Aβ40 (containing either the same FAD mutation, or wt‐Aβ40). The rate constants for this reaction were 1.8(1)×10^5^ m ^−1^ s^−1^, 1.96(9)×10^5^ m ^−1^ s^−1^ and 1.8(2)×10^5^ m ^−1^ s^−1^ for H6R, D7N and pS8 respectively (∼50, ∼60, and ∼50 % faster than for wt‐Aβ16 (1.22(8)×10^5^ m ^−1^ s^−1^); Figure 4). The rate enhancement for the two mutants seems to correlate well with the earlier onset of FAD (age between 55 and 60; sporadic AD mostly occurs after 85). The similar rate enhancement for phosphorylated Aβ correlates with the increased level of Aβ phosphorylation and aggregation in AD patients.

It is not clear if copper‐bridged dimer formation is the kinetic determinant of Aβ aggregation, or if dimerisation goes via two Cu^2+^‐bound Aβ monomers.^38^ A zinc‐bridged dimer was suggested recently to be a key intermediate in Aβ oligomerisation.^39^ If the former were true, the rate determined here would be an effective indicator. We note that under pseudo‐first‐order reaction conditions (with nanomolar Aβ**⋅**Cu^2+^, near physiological pH), the copper‐bridged heterodimer was not well populated, as fluorescence quenching was almost fully recovered. Nevertheless, this condition might be representative of that in the synaptic clefts of neurons. This means that the dissociation of the copper‐bridged dimer is much faster than its formation. Thus, the measured rate of heterodimer formation could also be regarded as the rate of copper exchange between the Aβ ligands. “Hopping” of redox‐active copper on the neuron membrane via Aβ might increase lipid and membrane receptor peroxidation mediated by Aβ.^40^ This process could also oxidise Aβ itself (e.g. di‐tyrosine crosslinking),^41^ thereby resulting in a stable covalently bonded Aβ dimer. As such, Cu^2+^‐enhanced reaction between mutant Aβs and between wt‐Aβ and post‐translationally modified Aβ might boost oligomer “seed” formation (the rate‐limiting step for fibril formation). Both copper‐bound Aβ monomer and copper‐bridged Aβ dimer have been proposed to be the intermediates towards aggregation.^32^, ^33^, ^42^, ^43^, ^44^ In order to unequivocally establish the oligomerisation mechanism in the presence of metal ions, Aβ peptides labelled with pairs of fluorophores would be necessary for the identification of co‐existing Aβ species. FRET or two‐colour coincidence detection at the single‐molecule level would allow direct measurement of time profiles for both copper‐bound Aβ monomer and copper‐bridged Aβ dimer.^45^, ^46^ However, mutations or post‐translational modifications on the N terminus of Aβ would not necessarily increase the reactivity of Aβ. Murine Aβ (which differs from human Aβ in three residues: R5G, Y10F and H13R) has higher binding affinity to Cu^2+^ but with much lower reactivity.^33^

## Conclusions

The kinetics of the interaction between copper and two Aβ mutants linked to FAD and one phosphorylated at Ser8 has been determined. The mutations and phosphorylation, which occur in the metal binding region, appear to have little effect on the rate at which Cu^2+^ binds to Aβ, even in the case of the English mutation (H6R), which lacks the coordinating His6 ligand. Dissociation rate, however, was affected by the mutations, thus resulting in different dissociation constants: *K*_d_ 2.1(2), 0.58(4) and 2.4(2) nm for H6R, D7N and pS8 respectively (wt‐Aβ: *K*_d_ 1.1(1) nm). The most interesting finding is that all three modified Aβ peptides have approximately 50 % faster second‐order reaction rates compared with wt‐Aβ, thus suggesting that copper‐assisted dimerisation of modified Aβ (formation of a copper‐bridged ternary complex) occurs considerably faster. In contrast, the thermodynamic dissociation constants appear not to correlate with the earlier age onset of AD, thus suggesting that kinetics could be the determinant for the roles of Aβ/metal‐ion interaction in the brain. Our quantitative investigation identified a potential molecular mechanism to explain the faster oligomerisation/fibrillisation of H6R and D7N Aβ mutants. Our results also suggests that phosphorylation of Aβ at Ser8 modulates its reactivity in a similar way as an FAD mutation, which may contribute to the aggregation of Aβ in the brain of sporadic AD patients.

## Experimental Section

**Materials**: HEPES (pH 7.5; Sigma–Aldrich) was used throughout as other buffers (e.g., phosphate buffers) are prone to precipitation in the presence of copper ions. Milli‐Q Ultrapure water (18.2 MΩ cm) was used throughout. A stock solution of NaCl (2 m in water; purity ≥99.5 %, Sigma–Aldrich) was stored at 5 °C. Copper(II) chloride dihydrate (purity >99 %, Sigma–Aldrich) was dissolved in water for a stock solution (20 mm) stored at 5 °C. This was diluted in HEPES (50 mm, pH 7.5) containing NaCl (100 mm) prior to experiments. Aβ peptides (AnaSpec) were purchased from Cambridge Bioscience (Cambridge, UK). Aβ_16_ samples were dissolved in HEPES (50 mm, pH 7.5) containing NaCl (100 mm) and stored at −20 °C. Aβ_40_ samples were dissolved in ammonium solution (1 %) to prevent aggregation and stored at −20 °C. All samples were diluted in HEPES (50 mm, pH 7.5) containing NaCl (100 mm) prior to experiments. Aβ_16_ peptides contained a fluorescent HiLyte 488 label at the C‐terminal lysine; Aβ_40_ peptides were unlabelled. The concentrations of stock solutions were determined by using a Lambda 25 UV/Vis Spectrometer (PerkinElmer). For labelled peptides, peak absorbance (*ϵ*=68 000 cm^−1^ m ^−1^) was used; unlabelled peptides were determined by absorbance at 280 nm (*ϵ*=1490 cm^−1^ m ^−1^).

**Stopped flow**: A HI‐TECH KinetAsyst SF‐61DX2 stopped‐flow spectrophotometer (TgK Scientific, Bradford‐on‐Avon, UK) was used at 298 K for kinetic measurements. Samples were excited by an MCLS1‐473‐20 fibre‐coupled laser diode (473 nm; Thorlabs, Newton, NJ). Prior to reaching the sample the excitation beam was split using a beam splitter to redirect the light (∼20 %) for use as a reference signal to account for fluctuations in laser intensity. Fluorescence emission was filtered through a 515GY filter (515 nm long pass; Comar Optics, Cambridge, UK) before detection in a photon multiplier tube. Data points were recorded with a log time‐scale sampling scheme. For each data point, a minimum of five traces were averaged. Time points below 2 ms were excluded because of instrument dead‐time (∼1 ms). The raw curves were fitted with exponentials in OriginPro (2015; OriginLab, Northampton, MA). The data points for the curves were weighted by using the time between each point, as an estimation of integration time. All error bars represent the uncertainty of the fit, propagated forward.

## Supporting information

As a service to our authors and readers, this journal provides supporting information supplied by the authors. Such materials are peer reviewed and may be re‐organized for online delivery, but are not copy‐edited or typeset. Technical support issues arising from supporting information (other than missing files) should be addressed to the authors.

Supplementary
