# Supplementary material for: Kinetics of the Interactions between Copper and Amyloid‐β with FAD Mutations and Phosphorylation at the N terminus
Source: Chembiochem. 2016 Aug 2;17(18):1732–7. doi: 10.1002/cbic.201600255 (PMC5096041; doi:10.1002/cbic.201600255)
Supplement: Supplementary file 1 — Supplementary [file CBIC-17-1732-s001.pdf]

CHEMBIOCHEM

Supporting Information

Kinetics of the Interactions between Copper and Amyloid-β with FAD Mutations and Phosphorylation at the N terminus

Paul Girvan,[b, c] Toru Miyake,[a, d] Xiangyu Teng,[b, c] Thomas Branch,[b, c] and Liming Ying*[a, b]

cbic_201600255_sm_miscellaneous_information.pdf

Supporting materials, methods & Figs S1, S2 & S3

Materials

The sequences of the various amyloid beta peptides used in this work are shown below.

Aβ16(H6R)	DAEFR	RDSGY	EVHHQ	K*					
Aβ16(D7N)	DAEFR	HNSGY	EVHHQ	K*					
Aβ16(pS8)	DAEFR	HDS†GY	EVHHQ	K*					
Aβ40(H6R)	DAEFR	RDSGY	EVHHQ	KLVFC	AEDVG	SNKGA	IIGLM	VGGVV	
Aβ40(D7N)	DAEFR	HNSGY	EVHHQ	KLVFC	AEDVG	SNKGA	IIGLM	VGGVV	
wt-Aβ40	DAEFR	HDSGY	EVHHQ	KLVFC	AEDVG	SNKGA	IIGLM	VGGVV	

* HiLyte 488 † phosphorylated serine

Coordination environments for Aβ · Cu^{2+} complexes

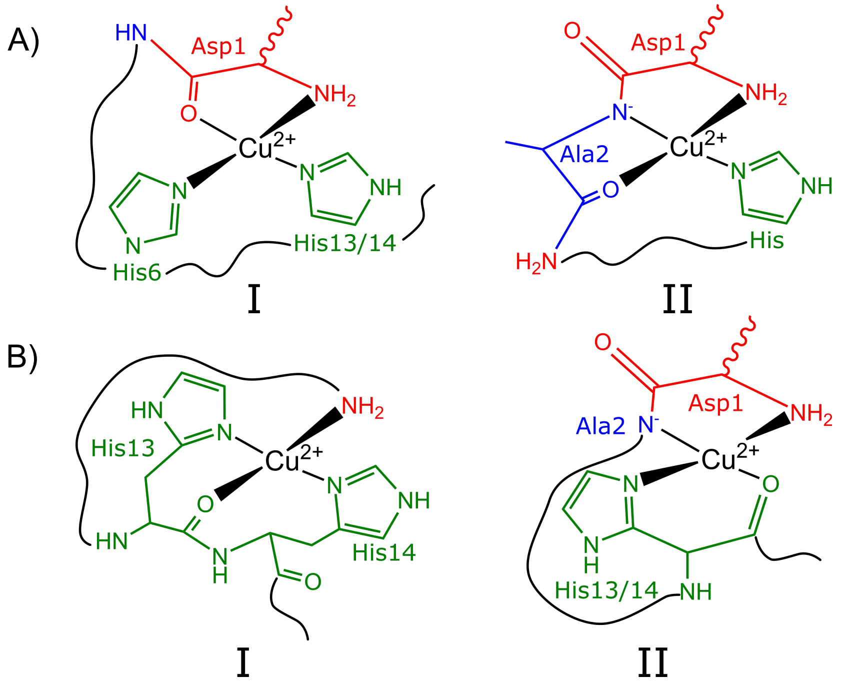

Figure S1 Coordination of Cu^{2+} bound to **A)** wt-Aβ and **B)** Aβ(H6R) showing both major components at physiological pH. Aβ(D7N) is expected to bind in a similar mannor to the wild type.[1]

Association rate constant of copper to Aβ

When determining the association rate of Aβ with Cu^{2+} independent of HEPES in the buffer, The HEPES concentration was varied from 10 mM to 100 mM, with 20 nM of Aβ and 400 nM of $CuCl_2$ in 100 mM NaCl. The apparent rate was obtained by fitting the fluorescence quenching data (Figure S2) with multi-exponentials, taking the major, fast phase. To determine the HEPES independent reaction rate, an empirically chosen parabola centred on zero was used to fit a plot of inverse apparent rate, against HEPES concentration, where the inverse of the intercept corresponds to the HEPES independant rate.

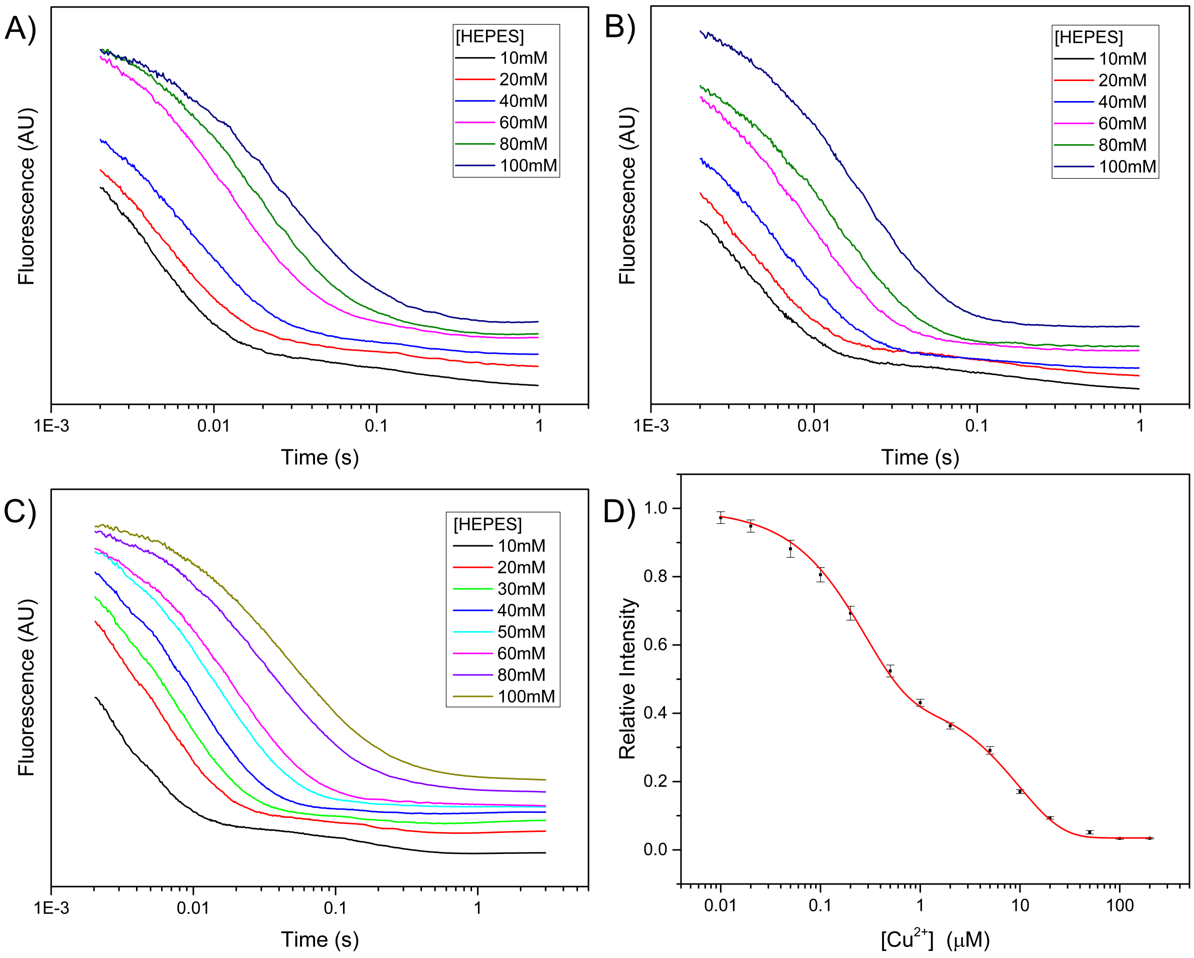

Figure S2 Kinetic traces showing quenching of fluorescence as Cu(II) binds to Aβ16 **A)**H6R, **B)**D7N and **C)**pS8 at various concentrations of HEPES. **D)** Fluorescence titration of 133 nM Hilyte 488 labelled Aβ16 by Cu^{2+} in 50 mM HEPES and 100 mM NaCl buffer solution (pH 7.5).

Interconversion of mutated (H6R & D7N) and phosphorelated pS8 Aβ·Cu complexes

The methods used were described in detail in the supporting information of our recent publication (Ref 2). Briefly, the minimum symmetric reaction scheme shown below was used to account for the spontaneous dissociations of the two Aβ·Cu species (Component **I** and **II**), their interconversion, and the reactions of the ligand (EDTA), with the two species.

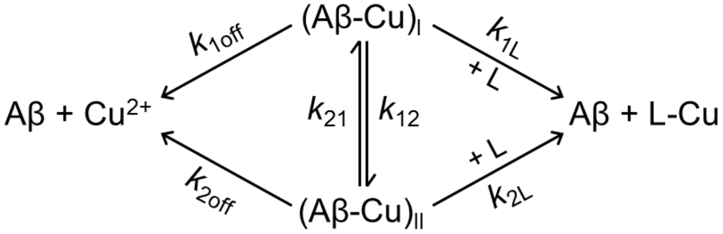

This model gives the following differential equations.

$$\frac{\mathrm{d}[(\mathrm{A\beta{\cdot}Cu})_{\mathrm{I}}]}{\mathrm{d}t} = -[(\mathrm{A\beta{\cdot}Cu})_{\mathrm{I}}](k_{1\mathrm{off}} + k_{1\mathrm{Loff}}[\mathrm{L}] + k_{1\to 2}) + [(\mathrm{A\beta{\cdot}Cu})_{\mathrm{II}}]k_{2\to 1} \tag{1}$$

$$\frac{\mathrm{d}[(\mathrm{A\beta{\cdot}Cu})_{\mathrm{II}}]}{\mathrm{d}t} = -[(\mathrm{A\beta{\cdot}Cu})_{\mathrm{II}}](k_{2\mathrm{off}} + k_{2\mathrm{Loff}}[\mathrm{L}] + k_{2\to 1}) + [(\mathrm{A\beta{\cdot}Cu})_{\mathrm{I}}]k_{1\to 2} \tag{2}$$

As the experiments here were performed under pseudo-first-order conditions we can assume

$$\frac{\mathrm{d}[\mathrm{L}]}{\mathrm{d}t} = 0 \tag{3}$$

An analytical solution can be found for the concentration of the two Aβ·Cu species as a function of time. The solutions are double exponential functions with amplitudes (A_1, A_2) and rate constants (k_1, k_2) as the following

$$A_1 = \frac{1}{2\delta}((\beta+\gamma)[(\mathrm{A\beta{\cdot}Cu})_{\mathrm{I}}]|_{t=0} - 2k_{2\to 1}[(\mathrm{A\beta{\cdot}Cu})_{\mathrm{II}}]|_{t=0} + (\beta-\gamma)[(\mathrm{A\beta{\cdot}Cu})_{\mathrm{II}}]|_{t=0} - 2k_{1\to 2}[(\mathrm{A\beta{\cdot}Cu})_{\mathrm{I}}]|_{t=0}) \tag{4}$$

$$k_1 = \frac{\alpha+\beta}{2} \tag{5}$$

$$A_2 = \frac{1}{2\delta}((\beta-\gamma)[(\mathrm{A\beta{\cdot}Cu})_{\mathrm{I}}]|_{t=0} + 2k_{2\to 1}[(\mathrm{A\beta{\cdot}Cu})_{\mathrm{II}}]|_{t=0} + (\beta+\gamma)[(\mathrm{A\beta{\cdot}Cu})_{\mathrm{II}}]|_{t=0} + 2k_{1\to 2}[(\mathrm{A\beta{\cdot}Cu})_{\mathrm{I}}]|_{t=0}) \tag{6}$$

$$k_2 = \frac{\alpha-\beta}{2} \tag{7}$$

Where

$$\alpha = k_{1\to2} + k_{2\to1} + k_{1\mathrm{off}} + k_{2\mathrm{off}} + (k_{1\mathrm{Loff}} + k_{2\mathrm{Loff}})[\mathrm{L}] \tag{8}$$

$$\beta = (k_{1\to2}^2 + 2k_{1\to2}(k_{2\to1} + k_{1\mathrm{off}} - k_{2\mathrm{off}} + (k_{1\mathrm{Loff}} - k_{2\mathrm{Loff}})[\mathrm{L}]) + (k_{2\to1} - k_{1\mathrm{off}} + k_{2\mathrm{off}} - (k_{1\mathrm{Loff}} - k_{2\mathrm{Loff}})[\mathrm{L}])^2)^{\frac{1}{2}} \tag{9}$$

$$\gamma = k_{1\to2} - k_{2\to1} + k_{1\mathrm{off}} - k_{2\mathrm{off}} + (k_{1\mathrm{Loff}} - k_{2\mathrm{Loff}})[\mathrm{L}] \tag{10}$$

$$\delta = \sqrt{-4(k_{2\to1}(k_{1\mathrm{off}} + k_{1\mathrm{Loff}}[\mathrm{L}]) + (k_{1\to2} + k_{1\mathrm{off}} + k_{1\mathrm{Loff}}[\mathrm{L}])(k_{2\mathrm{off}} + k_{2\mathrm{Loff}}[L])) + \alpha^2} \tag{11}$$

These solutions were used to fit the experimentally obtained data (Figure S3) and extract the parameters shown in the scheme above.

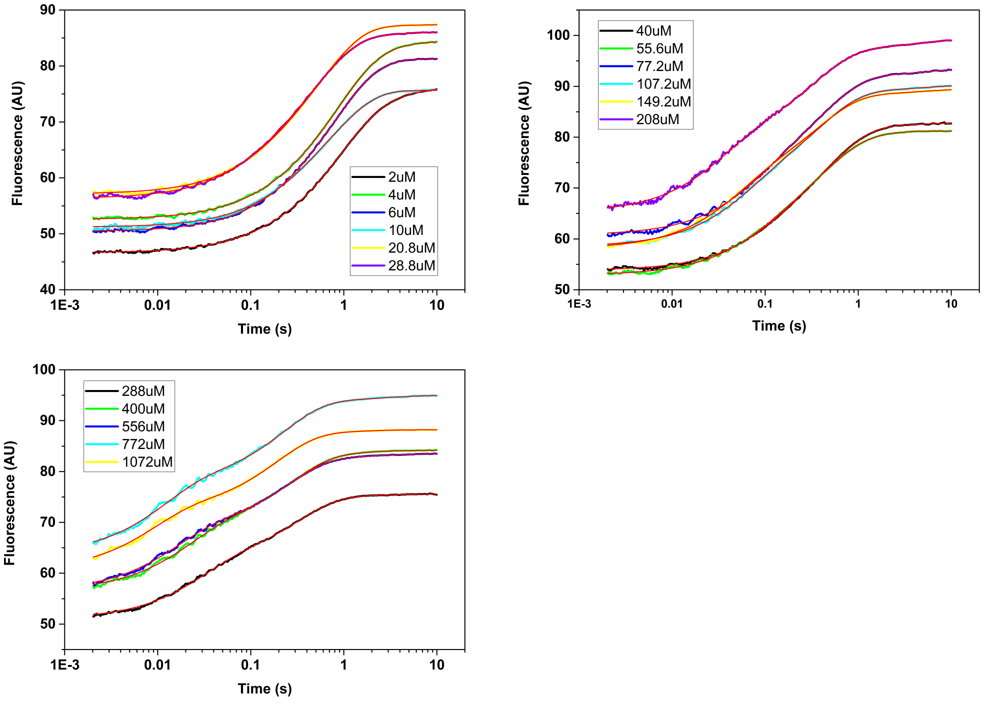

Figure S3a Raw kinetic traces showing recovery of fluorescence as Cu is removed from the Aβ(H6R)·Cu complex by various concentrations of EDTA. Fitted lines are shown in red.

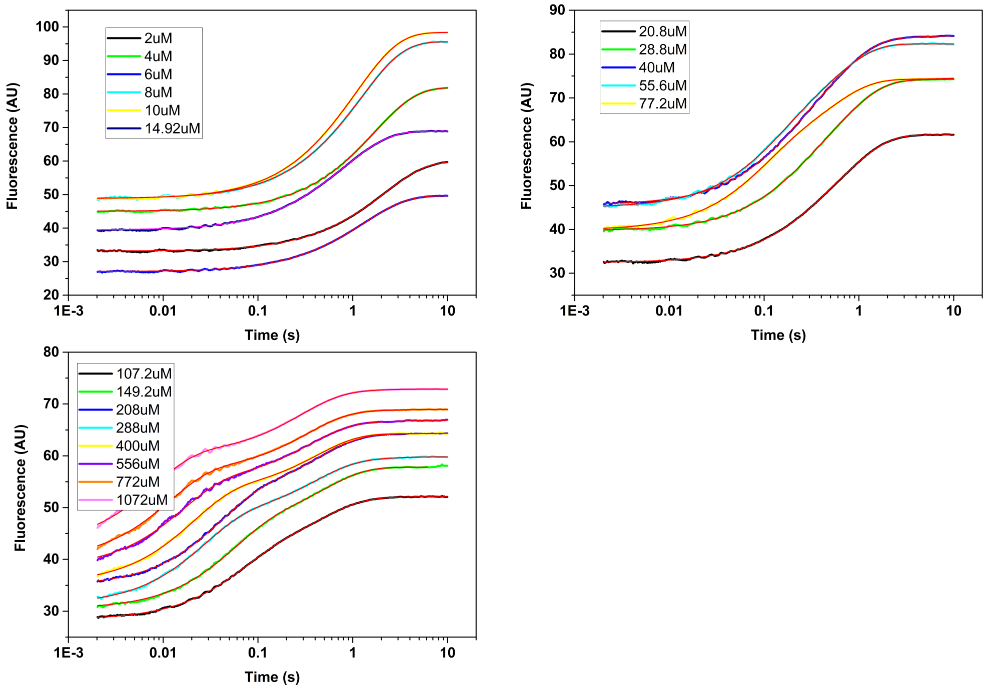

Figure S3b Raw kinetic traces showing recovery of fluorescence as Cu is removed from the Aβ(D7N)·Cu complex by various concentrations of EDTA. Fitted lines are shown in red.

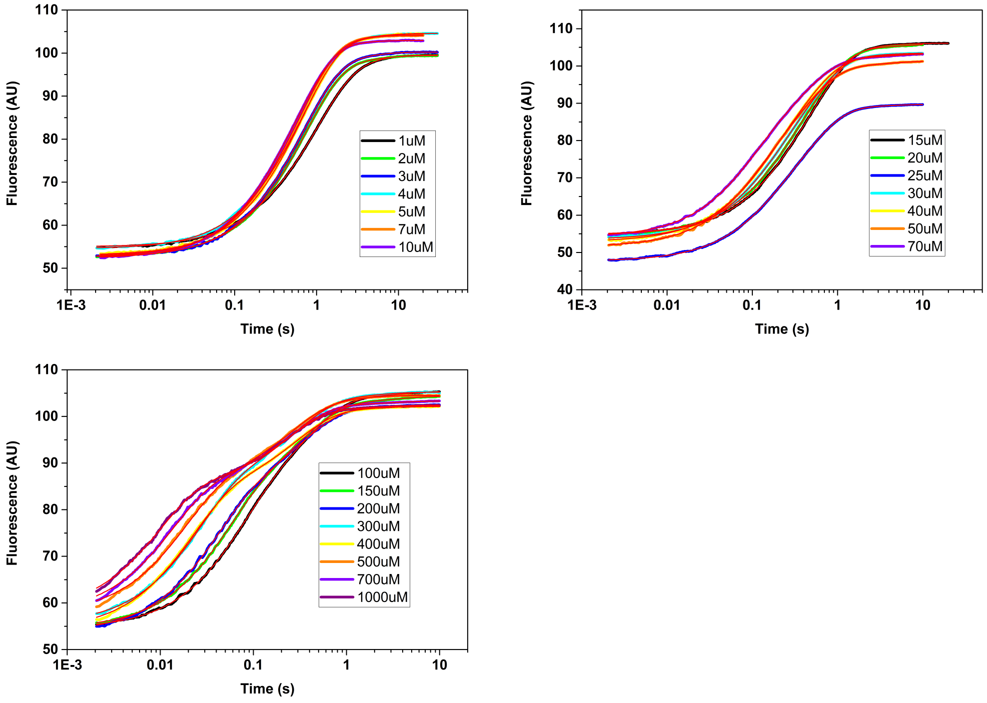

Figure S3c Raw kinetic traces showing recovery of fluorescence as Cu is removed from the Aβ(pS8)·Cu complex by various concentrations of EDTA. Fitted lines are shown in red.

Determing pK_a of mutated (H6R & D7N) and phosphorylated (pS8) Aβ·Cu complexes

The interconversion of Components **I** & **II** of the Aβ·Cu complex involves the removal of a proton. Therefor by using the rates of interconversion $k_{1\to2}$ & $k_{2\to1}$ determined from fitting the data to our model along with the Henderson–Hasselbalch equation the pK_a of Aβ·Cu complex can be determined.

$$(\mathrm{A\beta{\cdot}Cu})_\mathrm{I} \underset{k_{1\to2}}{\overset{k_{2\to1}}{\rightleftarrows}} (\mathrm{A\beta{\cdot}Cu})_\mathrm{II} + \mathrm{H}^+$$

$$\mathrm{pH} = \mathrm{p}K_\mathrm{a} + \log_{10}\frac{k_{1\to2}}{k_{2\to1}} \tag{12}$$

Derivation of apparent equilibrium dissociation constant of Aβ·Cu complex from kinetic rate constants

$$\mathrm{A\beta} + \mathrm{Cu}^{2+} \underset{k_\mathrm{off}}{\overset{k_\mathrm{on}}{\rightleftarrows}} (\mathrm{A\beta.\,Cu})_\mathrm{I} \underset{k_{2\to1}}{\overset{k_{1\to2}}{\rightleftarrows}} (\mathrm{A\beta.\,Cu})_\mathrm{II}$$

As the dissociation rate constant of Component **II** is much slower compared to that of the Component **I** and the interconversion rates, we can use the scheme shown above to work out the apparent equilibrium dissociation constant of Aβ·Cu complex.

$$K_\mathrm{A} = \frac{[(\mathrm{A\beta.\,Cu})_\mathrm{I}] + [(\mathrm{A\beta.\,Cu})_\mathrm{II}]}{[\mathrm{A\beta}][\mathrm{Cu}]} = \frac{k_\mathrm{on}}{k_\mathrm{off}}\left(1 + \frac{k_{1\to2}}{k_{2\to1}}\right) \tag{13}$$

$$K_\mathrm{D} = \frac{1}{K_\mathrm{A}} \tag{14}$$

Reference

1. Alies B., Eury H., Bijani C., Rechignat L., Faller P., Hureau C., pH-Dependent Cu(II) Coordination to Amyloid-βPeptide: Impact of Sequence Alterations, Including the H6R and D7N Familial Mutations, *Inorg. Chem.* **50**, 11192-11201, (2011).

2. Branch T., Girvan P., Barahona M., Ying L., Introduction of a fluorescent probe to amyloid-β to reveals kinetic insights into its interactions with Copper(II), *Angew. Chem. Int. Ed.* **54**, 1227-1230 (2015).
